# Methicillin Susceptible *Staphylococcus aureus* (MSSA) of Clonal Complex CC398, t571 from Infections in Humans Are Still Rare in Germany

**DOI:** 10.1371/journal.pone.0083165

**Published:** 2013-12-18

**Authors:** Christiane Cuny, Franziska Layer, Robin Köck, Guido Werner, Wolfgang Witte

**Affiliations:** 1 National Reference Center for Staphylococci and Enterococci, Robert Koch Institute, Wernigerode Branch, Wernigerode, Germany; 2 Institute of Hygiene, University Hospital Münster, Münster, Germany; 3 Robert Koch Institute, Wernigerode Branch, Wernigerode, Germany; University Hospital Münster, Germany

## Abstract

Methicillin-susceptible *Staphylococcus aureus* (MSSA) attributed to clonal complex (CC) 398 and exhibiting *spa*-type t571 received attention in Europe and in the USA for being associated with severe infections in humans. As this *spa*-type is exhibited by livestock-associated (LA) Methicillin-resistant *S. aureus* (MRSA) as well, it is important to discriminate LA- and human-derived strains by easy to perform, PCR-based methods. MSSA t571 contain phage int3 carrying *scn* and *chp*, whereas LA-MRSA t571 lack these markers. In contrast, pathogenicity island SaPIbov5 (detected by PCR bridging *vwb*bov and *scn*) is contained by LA-MRSA t571 and absent in the human MSSA subpopulation. Furthermore, MSSA t571 contain *erm*(T), the particular genomic arrangement of which was assessed by a PCR bridging *erm*(T) and the adjacent transposase gene. MSSA t571 are rare so far in Germany among isolates from infections in humans (0.14%) as well as among isolates from nasal colonization (0.13%). LA-MRSA t571 are also infrequent among MRSA isolated from carriage at admission to hospitals (0.1%) and also among isolates from infections in humans (0.013%).

## Introduction

Livestock-associated MRSA (LA-MRSA) attributed to clonal complex CC398 became prominent because of its wide dissemination as nasal colonizer of livestock such as pigs, veal calves, turkeys, and chicken in many countries in Europe, Northern America, and Asia [1]. It frequently colonizes the anterior nares of humans with occupational exposure to livestock in conventional farming systems [2], [3]. Human-to-human transmission beyond farms was also reported [2]. Due to its zoonotic potential, namely the capacity to cause zoonotic infections in humans, LA-MRSA belonging to clonal complex (CC) 398 as determined by multilocus sequence typing (MLST), became a public health concern: severe infections of skin and soft-tissue, infections after joint replacement, cases of septicemia and even necrotizing pneumonia in pig farmers and their family members but also in humans without direct contact to livestock were reported [4], [5], [6]. LA-MRSA CC398 were also introduced into hospitals and cause nosocomial infections such as postoperative wound infections and ventilator-associated pneumonia [6]. Its capacity to further disseminate in these settings appears to be low so far [7].

MSSA CC398, exhibiting *S. aureus* protein A (*spa*) type t571 were already reported from pigs and from farmers in France in 2005 [8]. During the past 3 years reports on invasive infections in humans by MSSA t571, in particular septicemia, from France [9], [10], and from Belgium [11] attracted attention. Although hitherto at low level (proportion of 2.2% among the sample of MSSA blood culture isolates), a sevenfold increase was recorded in France (10). MSSA t571 were also reported from bloodstream (BSI) infections in humans with no animal contacts in a Northern Manhattan community [12] where they had been described as nasal colonizers too [13]. At first sight, *spa* typing and attribution of types to the MLST CC398 suggest a zoonotic origin for MSSA t571 isolates detected among humans. However, genomic-based analysis revealed particular characteristics of isolates from BSI infections in humans which were not seen in LA-MRSA CC398 so far and suggested a human-adapted subpopulation in *S. aureus* CC398 [14], [15]. Usually *S. aureus* from pigs and from ruminants lack the immune evasion gene cluster which is phenotypically indicated by β-hemolysis on sheep blood agar. If complete, this gene cluster includes genes *sak*, *chp*, and *scn* and is contained by prophages of integrase type 3 (*int*3) which integrate into *hlb* coding for β-hemolysine [16]. Differently from LA-MRSA CC398, most MSSA CC398, t571 from humans possesses *int*3-phages, containing *chp* and *scn* but not *sak*. Isolates exhibiting *spa* type t571 described so far were resistant to macrolides due to *erm*T mediated erythromycin resistance. This determinant was reported for streptococci and lactococci, and located on a plasmid as a separate replicon for an MRSA isolate CC398 of porcine and human origin too [17]. In MSSA t571 it was found to be located on a small plasmid which is integrated in the chromosome [15]. Thus, lack of β-hemolysis on sheep blood agar, PCR demonstration of *int*3, *chp*, and *scn*, as well as of chromosomally located *erm*T are targets for an easy to perform discrimination of MSSA t571 of human origin. As these characteristics were found in isolates from Northern America as well as from European countries a wide, international spread seems likely.

As reported for LA-MRSA CC398 from ruminants [18] and as also evident from the genomic sequences of LA-MRSA CC398, strain S0385 [15], possession of further alleles for the von-Willebrand factor binding protein (*vwb*) and staphylococcal complement inhibitor (*scn*) located on a particular pathogenicity island (SaPIbov5) [18] may represent host adaptation mechanisms. Therefore, demonstration of these determinants could indicate an animal origin of *S. aureus* CC398 exhibiting *spa* type t571.

Here we report on the prevalence of MSSA t571 among MSSA from colonization and from infections in humans in Germany and PCR-detectable markers for discrimination from MRSA CC398, t571 of animal origin.

## Materials and Methods

### Bacterial isolates

Discrimination between MRSA and MRSA was by oxacillin resistance phenotype. MSSA (n = 2890) and MRSA (n = 1361) isolates from various kinds of infections in humans originate from staphylococcal isolates which were sent to the German National Reference Center for staphylococci and enterococci for typing from 2006–2012. They were isolated from infections in 221 hospitals as well as in the community all over Germany. Furthermore, MRSA isolates (n = 3931) from infections in 39 hospitals and in the community in North-Western Germany, and from screenings at admission to the 39 hospitals in this geographical area which were typed at the Institute of Hygiene, University Hospital Münster (2008–2012; [19]) were included.

MSSA (n = 1486) isolates from nasal colonization of healthy humans in the community not admitted to hospitals originated from prospective studies performed in different parts of Germany (2006–2012), such as in the North-East of Germany [20], furthermore in Federal countries Brandenburg, Lower Saxony [2], and Saxony Anhalt.

### Molecular typing

Typing was performed for all isolates investigated; *spa* typing and grouping of staphylococcal chromosomal cassette *mec* (SCC*mec*) elements were performed as reported previously [2], for confirming the attribution of isolates exhibiting *spa* type t571 to MLST CC398 we used primers A07f/A07r and the PCR protocol as described by [21].

### PCR demonstration of resistance and virulence associated genes

These tests were performed for all isolates exhibiting t571. PCR for *mec*A, erm(A), erm(C), and *tet* genes (*tet*(K), *tet*(L), and *tet*(M) was performed according to (2), for *mec*C according to [22]. Primers for overlapping PCR covering both, *erm*(T) and the neighbouring transposase (*erm*(T)-*tpn*) gene were deduced from the genome sequence of strain ST398NM01 from the USA ([15]; acc.nr. CP003045): forward 5′GAATTCTTGTCTCTT, reverse 5′TATATCTGAAATAGTTCA. Conditions were: 95°C^5.00^ [95°C^0.30^; 50°C^30^; 72°C^30^]×35; 72°C^4.00^.

For PCR identification of phages possessing an integrase of type 3 we used primers int3,f2: 5′GTCAGCTTTAGATGACGC and int3,r2: 5′AGCGCTAATGATGAACGA according to NC_00227452. For PCR demonstration of *sak*, *chp*, and *scn* we followed the protocol according to [16].

Demonstration of genes *vwb* and *scn* as contained by SaPIbov5 was performed by PCR bridging both genes by use of the primer pairs scn,bov,f: 5′GATGAAGCTCTAGCTAAT, and vwb,bov,r: 5′CACAACGCTCCCAATGTT (sequences reduced from HM228919.1, conditions were 95°C^5.00^[95°C^0.30^; 50°C^30^; 72°C^30^]×35; 72°C^4.00^).

Demonstration of *luk*-PV by PCR was performed as described before [23].

### Antibiotic susceptibility testing (AST)

Phenotypical AST was performed according to the DIN laboratory standard (DIN EN ISO 20776-1 Labormedizinische Untersuchungen und In-vitro-Diagnostika-Systeme - Empfindlichkeitsprüfung von Infektionserregern und Evaluation von Geräten zur antimikrobiellen Empfindlichkeitsprüfung - Teil 1: Referenzmethode zur Testung der In-vitro-Aktivität von antimikrobiellen Substanzen gegen schnell wachsende aerobe Bakterien, die Infektionskrankheiten verursachen (ISO 20776-1:2006); Deutsche Fassung EN ISO 20776-1:2006.

## Results

In the following we arrange the results as shown in table 1 according to their MSSA/MRSA phenotypes and to their origin.

**Table 1:** Characteristics of MSSA and of MRSA^1^ exhibiting *spa* type t571 from humans and from animals.

Origin	No. total	No. isolates, t571 (year and origin)	β-hemolysis	PCR for													Resistance phenotype^2^
				A07	*mecA*	*int3*	*sak*	*chp*	*scn*	*ermT-tpn*	*ermA*	*ermB*	*ermC*	*tetM*	*vw-scn*	*luk-PV*	
Humans:																	
MSSA: blood cultures	433	2 (2010–2011)	−	+	−	+	−	+	+	+	−	−	−	−	−	−	PEN,ERY
MSSA: other infections	2457	1 (2006), wound	−	+	−	+	−	+	+	+	−	−	−	−	−	−	ERY
		1 (2009), abscess	−	+	−	+	−	+	+	+	−	−	−	−	−	−	ERY
MSSA: carriage, community	1486	1 (2012) nasal	−	+	−	+	−	+	+	+	+	−	+	−	−	−	ERY
		1 (2009) nasal	+	+	−	−	−	−	−	−	−	−	−	+	−	−	PEN,TET
MRSA: carriage, admission to hospitals	9414	1 (2009) nasal	−	+	−	+	−	+	+	+	−	−	−	−3	−	−	PEN,OXA,ERY,TET
		5 (2011–2012) nasal	+	+	+	−	−	−	−	−	+	−	+	+	+	−	PEN,OXA,ERY,CLI,TET
		4 (2008–2012) nasal	+	+	+	−	−	−	−	−	+	−	−	+	−	−	PEN,OXA,ERY,CLI,TET
		2 (2008) nasal	+	+	+	−	−	−	−	−	−	−	+	+	−	−	PEN,OXA,ERY,CLI,TET
		1 (2009) nasal	+	+	+	−	−	−	−	−	+	−	−	+	+	−	PEN,OXA,ERY,CLI,TET
		1 (2009) ear	+	+	+	−	−	−	−	−	+	−	+	+	+	−	PEN,OXA,ERY,CLI,TET
		1 (2008) throat	+	+	+	−	−	−	−	−	−	−	−	+	−	−	PEN,OXA,TET
MRSA: infections	1592	1 (2009) wound	+	+	+	−	−	−	−	−	+	−	−	+	+	−	PEN,OXA,ERY,CLI,TET
		1 (2012) wound	+	+	+	−	−	−	−	−	+	−	−	+	−	−	PEN,OXA,ERY,CLI,TET
**Animals:**																	
MRSA: nasal carriage	724	7 (2006–2012) pig	+	+	+	−	−	−	−	−	+	−	−	+/−	+	−	PEN,OXA,ERY,CLI,TET
		1 (2009) veal calf	+	+	+	−	−	−	−	−	+	−	−	+	+	−	PEN,OXA,ERY,CLI,TET

1 MRSA were defined by oxacillin resistance phenotype according to DIN laboratory standard (DIN EN ISO 20776-1).

2 Antibiotic susceptibility testing was performed for benzylpenicillin (PEN), oxaxillin (OXA), gentamicin (GEN), erythromycin (ERY), clindamycin (CLI), tetracycline (TET), cotrimoxazol (TMP), rifampicin (RIF), fusidic acid (FUS), fosfomycin (PHO), linezolid (LNZ), tigecycline (TIG), daptomycin (DAP), vancomycin (VAN), teicoplanin (TPL), mupirocin (MUP).

3 Also PCR negative for *tet*(K), *tet*(L), *tet*(M).

### MSSA t571 among isolates from infections in humans

Only 2 of 433 (0.46%) blood culture isolates and 2 of 2457 (0.08%%) isolates from other kinds of infection exhibited t571.For these 4 isolates presence of *int*3, *chp*, *scn*, and *erm*T was demonstrated by PCR. The arrangement of *erm*(T) neighboring a transposase gene was confirmed by a PCR covering both genes. PCR for SaPI5bov markers was negative. In the following we refer to this patterns as MSSA t571, *int*3 (*chp*, *scn*), *erm*(T). Phenotypic and genotypic antibiotic resistance patterns are shown in table 1.

### MSSA t571 among isolates from nasal colonization of humans

Two of 1486 isolates from nasal colonization of humans in the community exhibited t571 (0.13%). One of them originating from 2009 belonged to the *int*3 (*chp*, *scn*), *erm*(T) subpopulation, it was resistant to tetracycline, PCRs for *tet*(M), *tet*(K), and *tet*(L) were negative. The other isolate originated from a nasal swab from a veterinarian. It was β-hemolytic on sheep blood agar and PCR-negative for *int*3, *chp*, *scn*, and also for *erm*(T), but positive for SaPIbov5 markers as well as for *tet*M. A further isolate was *int*3 (*chp*, *scn*), *erm*(T) and originated from a nasal swab from a premature newborn (not shown in table 1).

### MRSA t571 from humans

Of particular interest were MRSA isolates from infections and colonization in humans. Theoretically they could originate from MSSA t571 *int*3 (*chp*, *scn*), *erm*(T) by acquisition of a *mec* gene. Only 2 of 15292 (0.018%) MRSA isolates from infections in humans exhibited t571. Both isolates contained *mec*A associated with SCC*mec*V as well as *erm*(A), and/or *erm*(C) but were negative for *int*3 and *erm*(T). The characteristics of these isolates correspond to those which are typical for LA-MRSA CC398 (table 1).

Among 9414 isolates from nasal carriers screened at admission 15 exhibited t571. One of these corresponded to MSSA t571 *int*3 (*chp*, *scn*), *erm*(T). It was resistant to oxacillin (MIC 4 mg/l); however, PCR for *mec*A, *mec*C as well as for SCC*mec* was negative. This isolate was resistant to tetracycline, PCR for *tet*(K), *tet*(L), and *tet*(M) was negative. The other MRSA t571 isolates exhibited characteristics which are typical for LA-MRSA CC398 such as lack of *int*3 and of *erm*(T), and possession of the “bovine” polymorph of *vwb*.

### MRSA t571 among MRSA isolates attributed to CC398 from livestock and from horses

724 MRSA isolates of animal origin were attributed to CC398 by *spa* types. Among them, 7 isolates from nasal swabs taken from pigs and 1 from a nasal swab taken from a veal calf exhibited t571 (8/724 = 1.1%). These 8 isolates were β-hemolytic and PCR-negative for *int*3, *chp*, *scn*, and also for *erm*(T), but positive for SaPIbov5 markers and PCR-positive for *tet*(M), (table 1).

## Discussion

Molecular typing-based surveillance of *S. aureus* mainly focuses on MRSA. As shown here MSSA also need attention in order to follow the emergence of strains with pronounced capacity to cause invasive infections such as MSSA t571, *int*3 (*chp*, *scn*), *erm*(T). In the retrospective sample from Germany described here the first isolate was observed in 2006. Its prevalence remained low so far (0.004–0.3% for isolates from infections and 0.13% for isolates from nasal carriage). MSSA t571 were also not abundant among MSSA isolates from 17 European countries collected in 2008 [24]. One of the MSSA t571 isolates from nasal colonization of a veterinarian identified in this study was lacking *int*3 (*chp*, *scn*), *erm*(T) and corresponded to LA-MRSA CC398 besides susceptibility to oxacillin. Hence, it probably lost *mec*A.

The low prevalence of MSSA t571, *int*3 (*chp*, *scn*), *erm*(T) is different from the situation in France where a prevalence of 2,3% was reported for MSSA from bloodstream infections affecting patients in 31 healthcare institutions in 3 different geographical areas from 2007 until 2010 [10]. A more recent study from a Manhattan-based hospital reported a prevalence of 3.2% among MSSA BSI isolates. Patients affected were more likely Hispanics living in the vicinity of this hospital [25]. These observations indicate that MSSA t571, *int*3 (*chp*, *scn*), *erm*T) has the potential for further dissemination once introduced as already suggested by comparative typing of isolates from Manhattan and the Dominican Republic [13]. The emergence of MSSA t571, *int*3 (*chp*, *scn*), *erm*(T) in neighbouring countries such as France (10) and Belgium (11) calls for timely recognition of dissemination in Germany. Besides *spa* type t571 other markers are desirable for rapid identification of human associated MSSA t571 as LA-MRSA CC398, t571 may lose methicillin resistance. As known from a number of previous studies, LA-MRSA CC398 in general are not only capable of colonizing humans but also to cause infections, in particular those of the skin and soft tissue [26], [27]. MRSA exhibiting t571 were very rare among MRSA isolates from infections in humans and also infrequent among our sample of isolates from animals (1%) as it was also reported from another study in Germany [28]. The low prevalence among human carriers (0.14%) corresponds to these data. With the exception of one isolate (MRSA, negative for *mec*A and *mec*C) the few MRSA t571 isolates from nasal colonizations and from infections in humans correspond to isolates of animal origin with respect to lack of the immune evasion gene cluster.

In LA-MRSA t571macrolide resistance is usually conferred by *erm*A, in part by additional possession of *erm*(B) and *erm*(C) but not by *erm*(T) so far. Furthermore, they are resistant to tetracycline and contain *tet*M which was not observed in MSSA t571, *int*3 (*chp*, *scn*), *erm*(T) so far.

One suitable marker for is the possession of the immune evasion gene cluster in the configuration typical for MSSA t571 (lack of *sak*). The immune evasion gene cluster is usually associated with *S. aureus* of human origin [29] as originally indicated by the finding of fibrinolytic activity of *S. aureus* from humans but not from animals [30]. Although described for LA-MRSA CC398 other than t571 from infections in humans [9] it is obviously no prerequisite to the capacity of isolates of primary animal origin to cause invasive infections in humans: as we found it in only 14% of MRSA CC398 human isolates originating from different kinds of infection (own unpublished data). Its role in adaptation to humans needs further exploration, and care should be taken when using it as a marker for pathogenicity to humans. Although *erm*(T) has been reported from LA-MRSA CC398 [31] it has not been described for MRSA t571 and can be used as an additional indicator when detected in MSSA t571 containing the immune evasion gene cluster (*int*3 [*chp*, *scn*]). The determinant for the bovine von Willebrand factor *vwb* associated with the *scn* gene when located on SaPIbov5 is contained only by 7 of the 15 MRSA t571 isolates investigated and therefore of limited value.

Besides MSSA t571 another group of MSSA which are attributed to CC398 and most probably related to the ancestral subpopulation attracted attention as causative agent of severe skin and soft tissue infections, these isolates contain *luk*PV as well as the immune evasion gene cluster and seem to be more widely disseminated in China [32]. Among the sample of 2890 MSSA isolates from infections in humans only 0.2% were attributed to CC398 and contained *luk*-PV as well as the immune evasion gene cluster. Two of them originated from different locations in 4 different German federal states and exhibited *spa* types t034 and t3625 (own unpublished data).

MSSA attributed to CC398 and capable of causing severe infections in humans are another example for the importance of establishing a DNA sequence-based first-line typing tool such as *spa* typing (for overview see [33]), and to supplement it with easy to perform tests for detection of subpopulations of particular significance.

Although there is an impressive and rapid progress in the development of whole genome based molecular typing of bacterial and viral pathogens [34], [35], implementation in molecular surveillance systems will need its time. Currently, we have to use accessible methods in a rational way.

## References

[1] Smith TC, Pearson N. (2011) The emergence of *Staphylococcus aureus* ST398. Vector Borne Zoonotic Dis. 11: , 327–39. 10.1089/vbz.2010.0072 20925523

[2] Cuny C, Nathaus R., Layer F, Strommenger B, Altman D, et al. (2009) Nasal colonization of humans with methicillin-resistant *Staphylococcus aureus* (MRSA) CC398 with and without exposure to pigs. PLoS One. 4(8): , 46800. 10.1371/journal.pone.0006800 PMC2728842 19710922

[3] van Cleef BA, Verkade EJ, Wulf MW, Buiting AG, Voss A. (2010) Prevalence of livestock-associated MRSA in communities with high pig-densities in The Netherlands. PLoS One 5(2): , e9385. 10.1371/journal.pone.0009385 PMC2828479 20195538

[4] Witte W, Strommenger B, Stanek C, Cuny C. (2007) Methicillin-resistant *Staphylococcus aureus* ST398 in humans and animals, Central Europe. Emerg Infect Dis. 13: , 255–258. 10.3201/eid1302.060924 PMC2725865 17479888

[5] Van Cleef BA, Monnet DL, Voss A, Krziwanek K, Allerberger F, et al. (2011) Livestock-associated methicillin-resistant *Staphylococcus aureus* in humans, Europe. Emerg Infect Dis. 17(3): , 502–505. 10.3201/eid1703.101036 PMC3166010 21392444

[6] Köck R, Harlizius J, Bressan N, Laerberg R, Wieler LH, et al. (2009) Prevalence and molecular characteristics of methicillin-resistant *Staphylococcus aureus* (MRSA) among pigs on German farms and import of livestock-related MRSA into hospitals. Eur J Clin Microbiol Infect Dis. 28(11): , 1375–82. 10.1007/s10096-009-0795-4 PMC2772956 19701815

[7] Wassenberg MW, Bootsma MC, Troelstra A, Kluytmans JA, Bonten MJ. (2011) Transmissibility of livestock-associated methicillin-resistant *Staphylococcus aureus* (ST398) in Dutch hospitals. Clin Microbiol Infect. 17: , 316. 10.1111/j.1469-0691.2010.03260.x 20459436

[8] Armand-Lefevre L, Ruimy R, Andremont A. (2005) Clonal comparison of *Staphylococcus aureus* isolates from healthy pig farmers, human controls, and pigs. Emerg Infect Dis. 11: , 711–714. 10.3201/eid1105.040866 PMC3320358 15890125

[9] Van der Mee-Marquet N, et al. (2011) Emergence of unusual bloodstream infections associated with pig-borne-like *Staphylococcus aureus* ST398 in France. Clin Infect Dis. 52: , 152–153. 10.1093/cid/ciq053 21148535

[10] Valentin-Domelier AS, Girard M, Bertrand X, Violette J, François P, Donnio PY, et al. (2011) ST398 *Staphylococcus aureus* responsible for bloodstream infections: an emerging human-adapted subclone? PLoS One 6(12): ,e28369. 10.1371/journal.pone.0028369 PMC3230624 22163008

[11] Vandendriessche S, Kadlec K, Schwarz S, Denis O. (2011) Methicillin-susceptible *Staphylococcus aureus* ST398-t571 harbouring the macrolide-lincosamide-streptogramin B resistance gene erm(T) in Belgian hospitals. J Antimicrob Chemother. 66: , 2455–9. 10.1093/jac/dkr348 21868413

[12] Mediavilla JR, Chen L, Uhlemann AC, Hanson BM, Rosenthal M, et al.(2012) Methicillin-susceptible *Staphylococcus aureus* ST398, New York and New Jersey, USA. Emerg Infect Dis. 18: , 700–2. 10.3201/eid1804.111419 PMC3309677 22469250

[13] Bhat M, Dumortier C, Taylor BS, Miller M, Vasquez G, et al. (2009) *Staphylococcus aureus* ST398, New York City and Dominican Republic. Emerg Infect Dis. 15: , 285–7. 10.3201/eid1502.080609 PMC2657615 19193274

[14] Price LB, Stegger M, Hasman H, Aziz M, Larsen J, et al. (2012) *Staphylococcus aureus* CC398: host adaptation and emergence of methicillin resistance in livestock. MBio. 3(1): , 40305–11. 10.1128/mBio.00305-11 PMC3280451 22354957

[15] Uhlemann AC, Porcella SF, Trivedi S, Sullivan SB, Hafer C, Kennedy AD, et al.(2012) Identification of a highly transmissible animal-independent *Staphylococcus aureus* ST398 clone with distinct genomic and cell adhesion properties.MBio. 3(2): , e00027–12. 10.1128/mBio.00027-12 PMC3302565 22375071

[16] van Wamel WJ, Rooijakkers SH, Ruyken M, van Kessel KP, van Strijp JA. (2006) The innate immune modulators staphylococcal complement inhibitor and chemotaxis inhibitory protein of *Staphylococcus aureus* are located on beta-hemolysin-converting bacteriophages. J Bacteriol. 188: , 1310–5. 10.1128/JB.188.4.1310-1315.2006 PMC1367213 16452413

[17] Kadlec K, Schwarz S. (2010) Identification of a plasmid-borne resistance gene cluster comprising the resistance genes erm(T), dfrK, and tet(L) in a porcine methicillin-resistant *Staphylococcus aureus* ST398 strain. Antimicrob Agents Chemother. 54(2): , 915–8. 10.1128/AAC.01091-09 PMC2812154 20008780

[18] Viana D, Blanco J, Tormo-Más MA, Selva L, Guinane CM, et al. (2010) Adaptation of *Staphylococcus aureus* to ruminant and equine hosts involves SaPI-carried variants of von Willebrand factor-binding protein. Mol Microbiol. 77(6): , 1583–94. 10.1111/j.1365-2958.2010.07312.x 20860091

[19] Köck R, Schaumburg F, Mellmann A, Köksal M, Jurke A, et al (2013) (2013) Livestock-associated methicillin-resistant *Staphylococcus aureus* (MRSA) as causes of human infection and colonization in Germany. PLoS One. 8(2): e55040 doi: 10.1371 23418434 10.1371/journal.pone.0055040 PMC3572123

[20] Holtfreter S, Grumann D, Schmudde M, Nguyen HT, Eichler P, et al.. (2012) Clonal distribution of superantigen genes in clinical Staphylococcus aureus isolates. 10.1128/JCM.00204-07 PMC1951235 17537946

[21] van Wamel WJ, Hansenová Manásková S, Fluit AC, Verbrugh H, de Neeling AJ, et al. (2010) Short term micro-evolution and PCR-detection of methicillin-resistant and -susceptible *Staphylococcus aureus* sequence type 398. Eur J Clin Microbiol Infect Dis. 29(1): , 119–22. 10.1007/s10096-009-0816-3 PMC2797422 19795142

[22] Cuny C, Layer F, Strommenger B, Witte W. (2011) Rare occurrence of methicillin-resistant *Staphylococcus aureus* CC130 with a novel mecA homologue in humans in Germany. PLoS One. 6(9): , e24360. 10.1371/journal.pone.0024360 PMC3169590 21931689

[23] Witte W, Strommenger B, Cuny C, Heuck D, Nuebel U (2007) 12 Methicillin-resistant Staphylococcus aureus containing the Panton-Valentine leucocidin gene in Germany in 2005 and 2006. J Antimicrob Chemother. 60(6): 1258–63. 17934203 10.1093/jac/dkm384

[24] Grundmann H, Aanensen DM, van den Wijngaard CC, Spratt BG, Harmsen D, et al European Staphylococcal Reference Laboratory Working Group. (2010) Geographic distribution of *Staphylococcus aureus* causing invasive infections in Europe: a molecular-epidemiological analysis. PLoS Med. 7(1): , e1000215. 10.1371/journal.pmed.1000215 PMC2796391 20084094

[25] Uhlemann AC, Hafer C, A Miko B, G Sowash M, Sullivan SB, et al (2013) 9 Emergence of Sequence Type 398 as a Community- and Healthcare-Associated Methicillin-Susceptible Staphylococcus aureus in Northern Manhattan. Clin Infect Dis. 57(5): 700–3. 23728142 10.1093/cid/cit375 PMC3739468

[26] Pantosti A. (2012) Methicillin-Resistant *Staphylococcus aureus* Associated with Animals and Its Relevance to Human Health. Front Microbiol. 3: , 127. 10.3389/fmicb.2012.00127 PMC3321498 22509176

[27] Cuny C, Köck R, Witte W. (2013) Livestock associated MRSA (LA-MRSA) and its relevance for humans in Germany. Int J Med Microbiol. S1438-4221(13)00025-8. 10.1016/j.ijmm.2013.02.010 23607972

[28] Argudín MA, Fetsch A, Tenhagen BA, Hammerl JA, Hertwig S, et al. (2010) High heterogeneity within methicillin-resistant Staphylococcus aureus ST398 isolates, defined by Cfr9I macrorestriction-pulsed-field gel electrophoresis profiles and spa and SCCmec types. Appl Environ Microbiol. 76(3): , 652–8. 10.1128/AEM.01721-09 PMC2813030 20023093

[29] Cuny C, Friedrich A, Kozytska S, Layer F, Nubel U, et al. (2010) Emergence of methicillin-resistant *Staphylococcus aureus* (MRSA) in different animal species. Int J Med Microbiol. 300: , 109–117. 10.1016/j.ijmm.2009.11.002 20005777

[30] Meyer M. (1967) A proposal for subdividing the species *Staphylococcus aureus* Int J Syst Bacteriol. 17: , 387–389.

[31] Gómez-Sanz E, Kadlec K, Feβler AT, Zarazaga M, Torres C, et al (2013) (2013) Novel erm(T)-carrying multiresistance plasmids from porcine and human isolates of methicillin-resistant Staphylococcus aureus ST398 that also harbor cadmium and copper resistance determinants. Antimicrob Agents Chemother. Jul; 57(7): 3275–82 10.1128/AAC.00171-13. Epub 2013 Apr 29 23629701 PMC3697343

[32] Zhao C, Liu Y, Zhao M, Liu Y, Yu Y, et al (2012) Characterization of community acquired Staphylococcus aureus associated with skin and soft tissue infection in Beijing: high prevalence of PVL+ST398. PLoS One 7(6): e38577 10.1371/journal.pone.0038577. Epub 2012 Jun 6 22701673 PMC3368899

[33] Sabat AJ, Budimir A, Nashev D, Sá-Leão R, van Dijl JM, et al. on behalf of the ESCMID Study Group of Epidemiological Markers (ESGEM). (2013) Overview of molecular typing methods for outbreak detection and epidemiological surveillance. Euro Surveill. 18(4): , 20380. 10.2807/ese.18.04.20380-en 23369389

[34] Parkhill J, Wren BW. (2011) Bacterial epidemiology and biology–lessons from genome sequencing. Genome Biol. 12(10): , 230. 10.1186/gb-2011-12-10-230 PMC3333767 22027015

[35] Vogel U, Szczepanowski R, Claus H, Jünemann S, Prior K, et al. (2012) Ion torrent personal genome machine sequencing for genomic typing of Neisseria meningitidis for rapid determination of multiple layers of typing information. J Clin Microbiol. 50(6): , 1889–94. 10.1128/JCM.00038-12 PMC3372157 22461678

